# Methylene Blue for Refractory Shock Due to Acute Pancreatitis and Cholangitis

**DOI:** 10.7759/cureus.93018

**Published:** 2025-09-23

**Authors:** Eliana Alweis, Philip Riddle, Gerardo Carino

**Affiliations:** 1 Biochemistry, Brown University, Providence, USA; 2 Pulmonary and Critical Care, Brown University, Providence, USA

**Keywords:** acute pancreatitis, methylene blue therapy, methylene blue treatment, pulmonary critical care, refractory septic shock, vasopressor use

## Abstract

Methylene blue is emerging as a promising adjunctive therapy for refractory septic shock, enabling reduced vasopressor use and fewer intensive care and hospital days. We present the case of a 78-year-old woman with acute pancreatitis and cholangitis who was initially treated with antibiotics, IV fluids, and endoscopic retrograde cholangiopancreatography but had persistent shock with vasopressor requirements. She was then treated with methylene as a 1 mg/kg bolus, followed by another 1 mg/kg infusion over six hours, to reduce overall pressor requirements. She improved significantly within hours of receiving methylene blue, as evidenced by the weaning of vasopressor support. Upon receiving the culture results, antibiotics could be de-escalated. She continued to improve and was soon able to transfer out of the intensive care unit and was ultimately discharged. This case highlights the potential promise of early methylene blue administration in patients with refractory septic shock. It emphasizes that initiation of methylene blue may occur before confirmatory culture data is available.

## Introduction

In septic shock, circulatory and metabolic abnormalities in response to infection and inflammation contribute to increased morbidity and mortality [1]. Despite international guidelines and advances in critical care, the development of septic shock continues to result in poor outcomes in many patients [2]. Current management strategies focus on appropriate fluid resuscitation and vasopressor therapy to counteract the vasodilatory state [1,2]. Norepinephrine is the first-line agent for septic shock due to its potent alpha-1 adrenergic effects, which cause vasoconstriction and increase perfusion pressure [3]. There is great interest in non-catecholamine vasoactive medications (e.g., vasopressin) and other drugs to reduce the overall dose of required norepinephrine, thereby minimizing the side effects often associated with norepinephrine [4].

Methylene blue has been used for years in various medical applications, including the treatment of methemoglobinemia and as a medical dye, but has been more recently studied for its potential use as a rescue therapy for septic shock cases [4,5]. Methylene blue reduces circulating levels of NO, and therefore minimizes its vasodilatory effects in septic patients, potentially reducing overall vasopressor needs in these patients [6]. We present here a case of the early successful use of methylene blue in a patient with refractory shock due to cholangitis and acute pancreatitis. Methylene may have a role in treating.

## Case presentation

A 78-year-old female with a past medical history of multiple comorbidities, including chronic obstructive pulmonary disease, obstructive sleep apnea, and hypothyroidism, presented to the emergency department with one day of nausea, vomiting, and right upper quadrant pain that radiates to her back. She reported having symptoms intermittently for approximately one month, which worsened at the time of presentation. Lab results are presented in Table 1 and interpreted as most consistent with pancreatitis and with possible cholangitis.

**Table 1:** Lab values upon presentation with normal ranges WBC: white blood cell count, AST: aspartate aminotransferase, ALT: alanine aminotransferase, total bili: total bilirubin, ALP: alkaline phosphatase

	Lab value	Normal range
WBC	22.0/uL	4.0-11.0/uL
AST	538 IU/L	12-52 IU/L
ALT	334 IU/L	7-49 IU/L
Total Bili	2.4 mg/dL	0.1-1.2 mg/dL
ALP	334 IU/L	40-147 IU/L
Lipase	>3000 IU/L	0-160 IU/L

**Figure 1:**
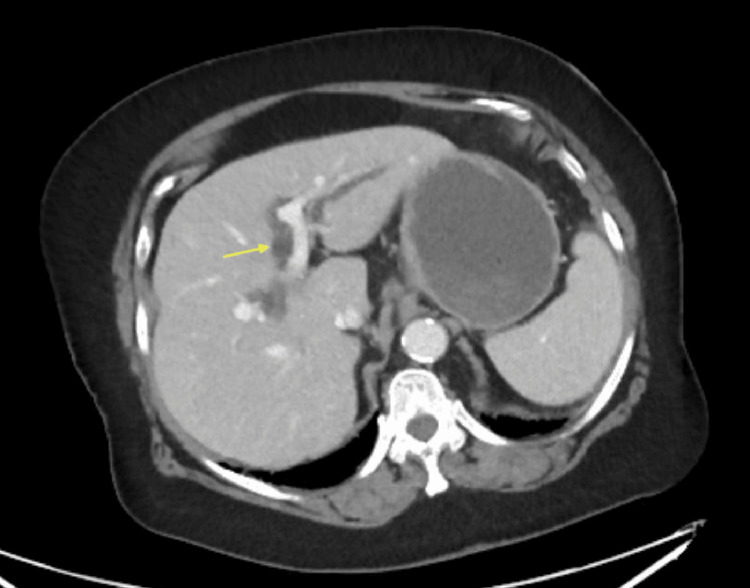
Axial abdominal CT scan showing intrahepatic bile duct dilated to >2.5 mm suggestive of biliary obstruction (yellow arrow) CT: computed tomography

**Figure 2:**
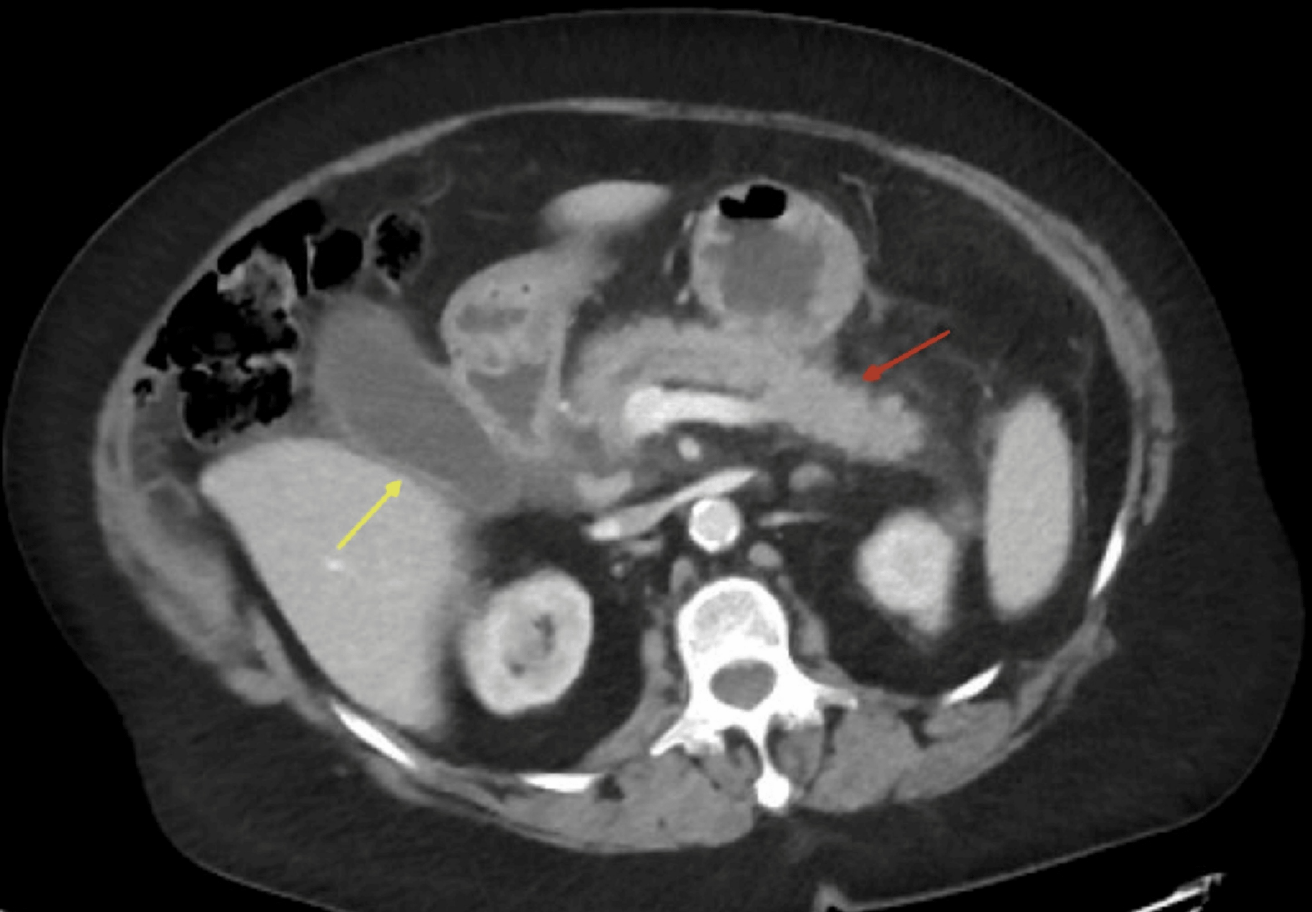
Axial abdominal CT scan showing the distended gallbladder with thickened wall (yellow arrow) and inflamed pancreas with peripancreatic stranding (red arrow) consistent with acute pancreatitis CT: computed tomography

A CT scan of the abdomen was performed, showing peripancreatic stranding, dilated intra- and extrahepatic bile ducts (Figure 1), and mild gallbladder wall thickening and pancreatic inflammation (Figure 2), suggestive of acute pancreatitis with possible biliary obstruction. She was admitted to the internal medicine service for acute pancreatitis and suspected cholangitis.

On hospital day 2, she developed acute hypoxic and hypercarbic respiratory failure and shock and was transferred to the intensive care unit. Endoscopic retrograde cholangiopancreatography (ERCP) showed biliary dilation, but no stones or pus, and a biliary sphincterotomy was performed with placement of a 10 French plastic stent. Post-procedure, it remained unclear if a biliary infection was the cause, as there were no supportive findings in ERCP. She required rapidly increasing doses of vasopressor support, including norepinephrine up to 40 mcg/min, vasopressin at 0.03 units/min, and epinephrine at 2 mcg/min, in addition to hydrocortisone 100 mg every eight hours, suggesting worsening and refractory shock. Due to this, methylene blue was administered as a 1 mg/kg bolus, followed by an additional 1 mg/kg infusion over six hours, to reduce overall pressor requirements. Within two hours of completing the methylene blue infusions, the vasopressors were weaned to single-digit norepinephrine support. This temporal response strongly suggests methylene blue’s contribution to hemodynamic recovery.

Blood cultures were obtained on hospital day 2 and were subsequently found to be positive for *Escherichia coli* and *Enterococcus faecalis* on hospital day 3, confirming bacteremia from the suspected biliary source. She was treated with vancomycin and piperacillin-tazobactam initially, but de-escalated to ampicillin-sulbactam when sensitivities returned as pan-sensitive. She continued to improve clinically, and three days post-methylene blue, she was completely weaned off vasopressors, extubated, and then transferred to the hospital medicine service. She was able to complete six more days of inpatient IV antibiotics and then transferred home on day 7 after methylene blue.

## Discussion

Acute pancreatitis is an inflammatory disease involving the exocrine pancreas, which results in abdominal pain and, in severe cases, may also lead to multiple organ dysfunction and vasodilatory shock [7]. Globally, incidence is 30-40 per 100,000, with mortality up to 15%, rising to 30% for multiorgan dysfunction [5,7]. In Europe and North America, cholelithiasis accounts for 20-70% of cases, with other causes including ethanol or drug use, hypertriglyceridemia, and ERCP. Patients can present with epigastric or diffuse abdominal pain, nausea and vomiting, abdominal distension, impaired consciousness, hypoxia, tachypnoea, tachycardia, and/or hypotension. According to the Atlanta classification, to diagnose a patient with acute pancreatitis, two of the three must be present: abdominal pain consistent with pancreatitis, serum amylase or lipase levels that are three or more times higher than the upper limits of normal, and a CT or MRI showing cross-sectional abdominal imaging consistent with pancreatitis [8]. There are several mechanisms through which pancreatitis affects the body, including diminished ATP production, increased intracellular calcium levels, and elevated levels of pro-inflammatory cytokines [7]. These effects can, in turn, disturb pancreatic and renal mitochondrial function, which can disrupt the electron transport chain and lead to altered nitric oxide production [5]. Nitric oxide synthases convert the amino acid L-arginine into nitric oxide, which subsequently activates the second messenger guanylate cyclase, resulting in vasodilation. There are two types of nitric oxide synthases: constitutive and inducible. The inducible form is produced upon activation by cytokines or endotoxins [4].

Important first-line treatments for acute pancreatitis include oxygen, fluid resuscitation, and pain management. Fluid resuscitation specifically can help correct third-space volume loss and tissue hypoperfusion, in addition to helping resolve organ dysfunction [7]. Vasodilatory effects play a significant role in the pathology of pancreatitis and can result in shock as a result of the organ dysfunction and hypotension caused [9]. Methylene blue can help reverse vasodilation by inhibiting iNOS, targeting cGMP, and possibly scavenging NO directly, aiming to treat pancreatitis and prevent the onset of septic shock [3]. In rat models, injection of methylene blue prior to induction of acute pancreatitis did not improve mitochondrial function in the pancreas. Still, it showed significant improvement in the kidneys and reduced vasodilation [5]. These benefits cannot be clearly extrapolated to humans, so further research is needed to explore the effects of methylene blue on human patients.

In cases of refractory septic shock, the administration of methylene blue results in a decreased time to vasopressor discontinuation, as well as shorter ICU and hospital lengths of stay [1,4]. Studies have yielded mixed results regarding mortality, with some finding no significant difference in mortality for patients treated with methylene blue compared to those not treated with this compound. In contrast, other studies have found lower mortality rates with the administration of methylene blue [1,4]. While methylene blue should be avoided in patients with G6PD deficiency due to the risk of causing hemolytic anemia [10], studies have consistently shown that methylene blue has no serious adverse effects, especially when used in doses of less than 2 mg/kg [1]. Various studies have tested doses ranging from 1 to 7 mg/kg, administered as boluses, continuous infusion, or a combination of both. Further work is needed to understand the pharmacokinetics of methylene blue to determine the optimal dose regimens. However, studies have found that the hemodynamic effects of methylene blue are short-lived and transient. This implies that appropriate treatment with methylene blue would likely involve either repeated boluses or continuous infusion.

Side effects of methylene blue treatment include possible elevated methemoglobin levels, but not to clinically significant levels, and transient blue discoloration of the skin or urine. It should also be noted that the pigment in methylene blue may interfere with pulse oximeter readings. In general, methylene blue is promising due to its safety, availability, and low cost [1]. However, the safety profile of methylene blue used at higher doses in septic shock patients is not clearly understood, as studies using it for shock have thus far involved small numbers of patients [11].

Furthermore, the use of methylene blue should be considered earlier in treatment, as it is possible to miss the optimal window of effectiveness. In this case, the patient was already in multipressor shock before methylene blue was initiated; however, no clear infectious source had been identified at that time. The shock from pancreatitis can occur with or without sepsis [4], so the decision to wait for confirmatory cultures before adding methylene blue might lead to dangerous delays. Shock is a very dangerous complication of acute pancreatitis. One study found it to be the underlying cause of fatality for 43% of patients who died as a result of acute pancreatitis [9]. This case highlights the potential role of early methylene blue administration in pancreatitis-associated shock, even prior to the confirmation of sepsis. The treatment had a significant effect, resulting in rapid weaning of vasopressors and a notable improvement in patient health overnight. As previous studies suggest that early use of methylene blue in septic shock may be advantageous, we would advocate that this case supports use in refractory shock, even before sepsis is clearly diagnosed.

## Conclusions

This report presents a case where the administration of methylene blue led to significant improvement of refractory shock in a 78-year-old woman with acute pancreatitis and cholangitis. A significant improvement occurred within hours of administering two doses of methylene blue over a six-hour period and included the rapid weaning of vasopressor support, ultimately leading to transfer out of the intensive care unit. This case suggests that early administration of methylene blue for refractoriness before a clear infectious source is identified may be beneficial, but further studies are needed before firm recommendations can be made. Questions regarding overall efficacy, dosing, and safety remain.

## References

[1] Ibarra-Estrada M, Kattan E, Aguilera-González P (2023). Early adjunctive methylene blue in patients with septic shock: a randomized controlled trial. Crit Care.

[2] Evans L, Rhodes A, Alhazzani W (2021). Surviving sepsis campaign: international guidelines for management of sepsis and septic shock 2021. Crit Care Med.

[3] Maheshwari N, Malkania B, Mudiyanselage R (2025). Methylene blue in septic shock: emerging evidence, clinical applications, and future directions. Cureus.

[4] Ng KT, Kwok PE, Lim WE, Teoh WY, Hasan MS, Zainal Abidin MF (2025). The use of methylene blue in adult patients with septic shock: a systematic review and meta-analysis. Braz J Anesthesiol.

[5] Kuliaviene I, Baniene R, Virketyte S (2016). Methylene blue attenuates mitochondrial dysfunction of rat kidney during experimental acute pancreatitis. J Dig Dis.

[6] Mayer B, Brunner F, Schmidt K (1993). Inhibition of nitric oxide synthesis by methylene blue. Biochem Pharmacol.

[7] Szatmary P, Grammatikopoulos T, Cai W (2022). Acute pancreatitis: diagnosis and treatment. Drugs.

[8] Banks PA, Bollen TL, Dervenis C (2013). Classification of acute pancreatitis--2012: revision of the Atlanta classification and definitions by international consensus. Gut.

[9] Isenmann R, Henne-Bruns D, Adler G (2003). Shock and acute pancreatitis. Best Pract Res Clin Gastroenterol.

[10] Sikka P, Bindra VK, Kapoor S, Jain V, Saxena KK (2011). Blue cures blue but be cautious. J Pharm Bioallied Sci.

[11] Arias-Ortiz J, Vincent JL (2024). Administration of methylene blue in septic shock: pros and cons. Crit Care.

